# Repurposing Renin–Angiotensin System Drugs for the Treatment of Audiovestibular Disorders

**DOI:** 10.3390/jcm15020743

**Published:** 2026-01-16

**Authors:** Grant Podhajsky, Kiran S. Marla, Alec P. Marticoff, Kenny Nguyen, Tanner Kempton, Sepehr Salehpour, Caden Duffy, Douglas M. Bennion

**Affiliations:** 1 Department of Otolaryngology, Head & Neck Surgery, University of Iowa, Iowa City, IA 52242, USA; 2 Department of Otolaryngology, Head & Neck Surgery, University of Arkansas for Medical Sciences, Little Rock, AR 72205, USA

**Keywords:** renin–angiotensin system, angiotensin II, angiotensin II type 1 receptor, sensorineural hearing loss, tinnitus, vertigo, oxidative stress, inflammation, blood-labyrinth barrier

## Abstract

Audiovestibular disorders arising from the inner ear (e.g., hearing loss, tinnitus, vertigo) are widely prevalent in the United States. Yet, medical treatments targeting the underlying pathology of these disorders remain scarce. The practice of repurposing FDA-approved drugs for new therapeutic indications has become increasingly common, offering a lower risk route to treatment development with fewer barriers to implementation, as safety profiles are already established. The renin–angiotensin system (RAS) is well known for its role in blood pressure and fluid balance, and its overactivation induces acute and chronic inflammation and oxidative stress. This review discusses existing evidence and proposed otoprotective mechanisms of RAS inhibition, specifically using angiotensin II type 1 receptor blockers (ARBs), which support the repurposing of these medications as novel treatments to affect the inner ear pathologies that underlay hearing loss, tinnitus, and vertigo.

## 1. Introduction

Auditory dysfunction of the inner ear is widely prevalent in the United States, with sensorineural hearing loss (SNHL) affecting almost two-thirds of those aged 70 years or older [1]. Tinnitus, which is the perception of sound without an external source (commonly described as ringing, humming, hissing, or roaring), has a significant negative impact on the quality of life in many sufferers and is reported by 10–15% of the adult population [2,3]. Vertigo is also common, with symptoms of vestibular dysfunction (e.g., positional or episodic dizziness, imbalance, or disequilibrium) reported by roughly 5% of people in a general population survey and experienced disproportionately more by women and by older adults [4].

Despite the nearly universal experience of these various forms of auditory dysfunction, therapeutic options and access to treatments targeting inner ear pathology are limited. For SNHL, the most common clinical treatment is to recommend amplification of sound through hearing aids, which are now available over the counter for patients with mild hearing loss and continue to be available by prescription for those with moderate and severe levels of hearing loss, often at significant out of pocket cost to the patient due to a general lack of insurance coverage. For those with severe to profound unilateral or bilateral SNHL, cochlear implantation is becoming widely available to those with sufficiently good health to undergo surgery and with capacity to participate in a prolonged course of post-implantation auditory rehabilitation. Aside from the guideline-recommended use of high-dose steroids given by mouth or via intratympanic injection in the rare cases of sudden sensorineural hearing loss, there are no proven treatments to restore natural acoustic hearing thresholds once lost. Treatments for tinnitus are focused on masking strategies using hearing aids and sound generators and on managing symptoms through cognitive behavioral therapy. Currently, there are no effective pharmacologic treatments for tinnitus [2]. Recent FDA de novo approval was granted for a device using bimodal neuromodulation through electrical tongue stimulation in conjunction with sound therapy, called the Lenire device (Neuromod Devices Ltd, Dublin, Ireland), but this has not been widely accepted or used in clinical practice [5]. Other alternative therapies, including acupuncture and massage, have historically not had high-quality studies performed and would benefit from research with larger sample sizes and longer follow-up times, but may provide relief for specific patient subsets, such as those with somatically modifiable tinnitus or temporomandibular joint disorder [6,7,8,9,10]. Treatment of vestibular (i.e., inner-ear related) vertigo is highly dependent on the putative diagnosis, with many treatments focused on non-pharmacologic measures like physical therapy with vestibular rehabilitation [11]. For diagnoses that assume dysregulated electrolyte concentrations and fluid pressure within the fluid compartments of the inner ear, a pathologic process known as endolymphatic hydrops, diuretic medications or histine analogs coupled with salt-restricted diet are recommended first-line therapies, which is discussed in more detail herein [12]. For persistent postural–perceptual dizziness (PPPD), medications such as antihistamines and benzodiazepines are sometimes used to treat symptoms, but do not target the underlying cause of dysfunction, and can have sedative side effects [4]. Due to the lack of effective pharmacologic therapies for these conditions, interest has been shown in identifying molecular pathways to modulate and improve inner ear dysfunction.

**Figure 1:**
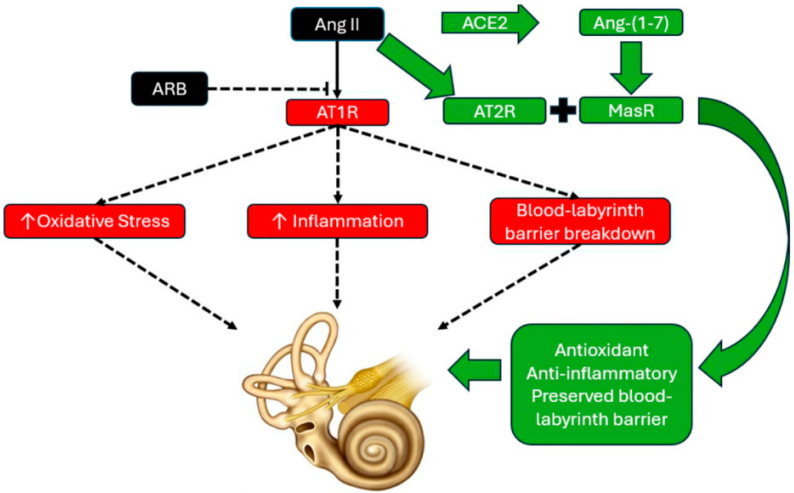
Diagram depicting angiotensin II (Ang II) type 1 receptor (AT1R blockade) with angiotensin receptor blocker (ARB) therapy. With ARB therapy, Ang II binds to the Ang II type 2 receptor (AT2R) and is also converted to angiotensin-(1-7) [Ang-(1-7)] through angiotensin converting enzyme 2 (ACE2). Once converted to Ang-(1-7), it activates the Mas receptor (MasR). Together, AT2R and Mas receptor activity promote antioxidant, anti-inflammatory effects, and are theorized to have preservative effects on the blood labyrinth barrier, as based on their protective effects on the blood–brain barrier. The combination of antioxidant, anti-inflammatory, and microvascular protection may form the basis for the otoprotective effects initially observed in published studies, warranting additional prospectively designed study of renin–angiotensin system (RAS)-targeted pharmacologic strategies as treatments for audiovestibular disorders.

The renin–angiotensin–aldosterone system or renin–angiotensin system (RAS) is well known for its effects on blood pressure, but it is also a key driver for both acute and chronic inflammation and oxidative stress [13,14]. Given its role in inflammation and oxidative stress, several model systems have sought to use RAS inhibition for anti-inflammatory, anti-oxidative, and vascular protective effects. Treatments targeting the RAS have been studied in many diseases with special relevance to the inner ear, including retinal vasculopathy, stroke, and vestibular schwannoma (VS) [15,16,17]. Given this context, we propose that RAS inhibition may be used for developing treatments for audiovestibular disorders affecting the inner ear (Figure 1). To accomplish this, we will describe the epidemiology, pathologic mechanisms, and current treatment options for the most common causes of sensorineural hearing loss (SNHL), tinnitus, and vertigo, followed by a review of emerging literature describing protective RAS-mediated effects, and concluding with a proposed mechanism for ongoing study of the RAS-targeted treatments for audiovestibular disorders. Given the emerging and heterogeneous nature of evidence regarding RAS-targeted therapies for audiovestibular disorders, we performed a non-systematic narrative review to synthesize preclinical and clinical findings. A formal systematic review with rigid inclusion criteria was not pursued, as our goal was to provide a broad overview and identify promising directions for future research.

## 2. Sensorineural Hearing Loss (SNHL)

### 2.1. Epidemiology of SNHL

SNHL, or hearing loss arising from impaired function of the sensory structures of the cochlear or the neural elements of the spiral ganglion and cochlear nerve, has several subtypes each with distinct prevalences and etiologies. The most common subtype of SNHL is presbycusis, or age-related hearing loss. Nearly two-thirds of individuals aged 70 years and older experience hearing loss, with over half affected by hearing loss severe enough to impair communication [1,18]. It is the third most common health condition affecting older adults after heart disease and arthritis [19]. The impact of presbycusis extends beyond hearing impairment and is associated with social isolation, depression, cognitive decline, and dementia [20].

Noise-induced hearing loss (NIHL) is the second most common form of hearing loss globally, affecting 5% of the population [21]. It is typically caused by repetitive exposure to loud noise from industrial, military, or recreational activities [21]. Noise exposure, especially at a young age, is also thought to hasten the progression of presbycusis [22]. Occupational hearing loss is the most prevalent work-related illness in the United States [23]. Studies estimate that approximately 22 million U.S. civilian workers are exposed to potentially damaging noise levels every year [24].

Certain medications are considered ototoxic and can also result in SNHL. According to data extracted from the FDA Adverse Event Reporting System between 2004 and 2023, there were over 100,000 adverse event reports of hearing impairment across 1300 different drugs [25]. Well-studied ototoxic medications include aminoglycoside antibiotics (e.g., amikacin, gentamicin, and tobramycin) and platinum-based chemotherapies (e.g., cisplatin and carboplatin) [26,27]. Among patients treated with aminoglycoside antibiotics for drug-resistant tuberculosis, the prevalence of ototoxic hearing loss is 41% [26]. For those receiving cisplatin and/or carboplatin chemotherapy, the prevalence of hearing loss is 43%, with cisplatin-only therapies reaching 49%, cisplatin and carboplatin combined at 56%, and carboplatin-only at 13% [27].

### 2.2. Pathophysiology of SNHL

Each subtype of SNHL possesses unique pathologic features, although some overlap exists among them. Presbycusis is a progressive, bilateral, and symmetrical sensorineural condition that results in decreased hearing thresholds at higher frequency sounds [18]. Presbycusis involves a combination of cochlear and neural degeneration, including loss of hair cells, striae atrophy, and spiral ganglion degeneration [18]. Oxidative stress from excessive reactive oxidative species (ROS) production can lead to mitochondrial DNA damage, and chronic low-grade inflammation drives the degenerative processes seen within the aging inner ear [28].

Genetic susceptibility also contributes to the pathophysiology of SNHL, particularly in presbycusis and congenital or early-onset forms. Variants affecting cochlear ion homeostasis, mitochondrial function, synaptic transmission, and oxidative stress response can increase vulnerability to age-related degeneration. Genetic predisposition may also modify susceptibility to environmental insults such as noise exposure and ototoxic medications, contributing to individual variability in disease severity [29].

Noise-induced hearing loss (NIHL) results from mechanical damage to cochlear structures, reduction in blood flow, sterile inflammation, oxidative stress, and glutamate excitotoxicity with resultant breakdown of synapses between hair cells and spiral ganglion neurons within the cochlea [30]. NIHL increases the production of ROS and reactive nitrogen species (RNS) derived from nitric oxide [31]. These ROS and RNS can persist within the cochlea for up to 1 week following the insult, triggering pro-apoptotic pathways that can lead to permanent hearing loss [30]. Immediately following acoustic overstimulation there are excessive levels of cytoplasmic calcium found in outer hair cells leading to mitochondrial damage, decreased energy, and eventual cell death [32]. In a noise-exposed environment, decreased capillary blood flow cannot supply enough oxygen and nutrients to maintain the ionic gradient that powers the endolymphatic potential necessary for normal cochlear function. The localized vascular constriction may also further disrupt cellular metabolism, leading to temporary or permanent shifts in auditory thresholds [33].

Drug-induced hearing loss from aminoglycosides and platinum-based chemotherapy is also thought to stem from the formation of excessive ROS, leading to oxidative stress, mitochondrial dysfunction, and activation of apoptotic pathways [31]. Most cases of SSNHL occur without an identifiable cause. Current theories suggest that SSNHL may occur in association with inflammatory pathways involving nuclear factor kappa B (NF-κB) activation, oxidative stress from excessive ROS, microthrombosis, viral infections, and metabolic diseases [28,34,35,36,37].

### 2.3. Current Treatments for SNHL

Given the proposed mechanism of oxidative stress as an underlying cause of SNHL, many studies have assessed medications and supplements with antioxidant properties. A recent meta-analysis suggests that N-acetyl cysteine may offer protective effects at low to mid frequencies in patients with NIHL, highlighting its potential as an antioxidant therapy [38]. The fat-soluble antioxidant coenzyme Q10, when deficient, has been associated with increased risk of idiopathic SSNHL, although no studies to date have demonstrated its efficacy in restoring hearing after onset [39].

Beyond traditional antioxidants, emerging observational data suggest that telmisartan, an angiotensin receptor blocker with partial peroxisome proliferator-activated receptor γ (PPARγ) agonist activity, is associated with a reduced incidence of hearing loss in hypertensive patients. Although this association has not been directly evaluated in the context of ototoxic diuretic use, the apparent protective effect appears unique to telmisartan among ARBs and is thought to reflect its partial PPARγ agonism rather than a class effect [40].

Adjunctive antioxidant therapy has shown promise in sudden SNHL management. A retrospective study found that high-dose vitamin C and vitamin E, when added to systemic steroids, improved both recovery rates and mean hearing gains [41]. Similarly, a prospective study demonstrated that supplementation with vitamins A, C, and E, along with selenium, significantly enhanced hearing outcomes compared to standard treatment alone [42]. Corticosteroids remain the cornerstone of sudden SNHL treatment, typically administered orally or via intratympanic injection within the first weeks after symptom onset [43]. The greatest benefit is observed when therapy begins within the first seven days [43]. However, a Cochrane review revealed mixed results, with two out of three randomized controlled trials showing no significant difference between steroid treatment and placebo [44].

To date, there are no FDA-approved medications to prevent or reverse NIHL. A systematic review and meta-analysis assessed corticosteroids, with or without hyperbaric oxygen therapy, for acute noise-induced hearing loss. Across five mostly retrospective studies, steroids alone improved hearing by about 6–9 dB, and combined therapy by 7–12 dB. However, results were inconsistent, treatment protocols varied, and key data were missing. No consensus guidelines exist, and the lack of control groups leaves spontaneous recovery rates uncertain [45]. In the absence of efficacious medical therapies, sound protection strategies with hearing protection devices (HPDs) like earplugs or earmuffs are recommended as an effective preventative intervention. Experimental studies have shown that earplugs are more effective at attenuating low-frequency noise, while earmuffs perform better at higher frequencies [46]. Although HPDs are effective, self-reported data from the National Health Interview Survey in 2007 and 2014 found that 53% of all noise-exposed workers reported non-use of HPDs [47].

Hearing aids are the primary treatment modality for mild (26–40 dB) to moderate (41–60 dB) SNHL, functioning by amplifying sound to improve communication. Despite their utility, nonadherence remains a concern, with 30–38% of patients discontinuing use due to perceived lack of benefit and device discomfort [48].

For cases of unilateral SNHL, such as in SSNHL, options include contralateral routing of sound (CROS) hearing aids or osseointegrated hearing implants, which are also an option for patients with anatomical deformities or intolerance to conventional hearing aids [49]. Cochlear implantation is recommended for individuals with severe (61–80 dB) to profound (≥81 dB) hearing loss and poor speech recognition (≤50–60%) despite optimal hearing aid use [50]. A scoping review of the literature from 2000 to 2018 reported substantial improvement in word perception scores, increasing from an average of 8.2% pre-implantation to 53.9% post-implantation [51].

### 2.4. RAS-Targeted Therapies for Hearing Loss

There has been a paucity of drug-discovery research across many inner ear disorders, and currently, there are no FDA-approved medications available for treating inner ear disorders. One way to advance the pharmacologic treatments of inner ear disorders is through drug repurposing studies. Drug repurposing studies have been previously utilized for inner ear disorders through exploring new treatments for SSNHL [52]. Researchers identified 42 candidate FDA-approved drugs that target genes expressed in either the spiral ganglion neurons or stria vascularis that were found to be steroid responsive in patients who were being treated for SSNHL. This approach provided a basis for establishing novel drug repurposing trials in acute otologic disorders [52]. Drug repurposing allows for faster development, lower cost, lower risk, and improved utilization of existing data and infrastructure.

Hypertension is a common condition that has a range of FDA-approved medications. ARBs are commonly prescribed as a first-line agent for the management of hypertension [53]. Additionally, hypertension, diabetes, and cardiovascular disease are known risk factors for the progression of hearing loss, making ARB therapy an ideal target for drug repurposing for the use of protection against inner ear disorders [54].

The underlying cause of audiovestibular disorders is often driven by inflammatory cascades and oxidative stress that lead to apoptotic and fibrotic pathways. The inflammatory cytokines (IL-1β, IL-6, TNF-α) produced after noise insults are also some of the same cytokines produced from AT1R activation [55,56]. Oxidative stress from excessive ROS is a known culprit driving audiovestibular disorders and is also produced when Ang II activates the AT1R [57,58].

Emerging pre-clinical and clinical studies suggest that RAS modulation with ARBs may reduce or prevent hearing loss. In a retrospective cohort study of 860,103 South Korean hypertensive patients, telmisartan use was associated with a significantly lower 3-year incidence of hearing loss (0.5% vs. 1.5%, *p* = 0.005) among 2193 patients who were taking Telmisartan when matched against those who were not [40]. Our own analysis of the *All of Us* database suggested that among >33,000 hypertensive patients with SNHL, those treated with ARBs or ACE-i were less likely to progress to cochlear implantation than those on calcium channel blockers or beta-blockers (Table 1) (queried in October 2022 by searching for patients with diagnostic codes for hypertension and cochlear implantation and stratified by first-line antihypertensive monotherapy). These findings should be interpreted with caution rather than as conclusive evidence, given the small number of cochlear implant cases and the inability to fully account for potential confounders such as genetic susceptibility, trauma, or other causes of hearing loss, and indicate that further prospective study is needed.

Another retrospective study evaluated patients with hypertension and vestibular schwannoma and found that those who were on ARB therapy were more likely to have normal baseline hearing and no progressive hearing loss during follow-up. Patients taking other anti-hypertensives showed declines that are consistent with the natural history of vestibular tumors. Further, RNAseq analyses of tissues from these patients that defined a core losartan response gene signature for tumor associated macrophages revealed a downregulation of multiple genes associated with inflammation, fibrosis, and hearing loss-related pathways [59]. Another similar retrospective study revealed slightly less hearing decline among 26 patients with vestibular schwannomas who were taking losartan, though this did not reach statistical significance, highlighting the need for higher powered prospective studies with longer-term follow-up [60].

Preclinical studies have similarly demonstrated RAS-modulated protection against hearing loss. In a murine model of Alport syndrome, mice treated with Sparsentan (a single molecule dual endothelin type-A and angiotensin II type 1 receptor antagonist) prevented the accumulation of extracellular matrix in the strial capillary basement membranes in the inner ear and reduced susceptibility to hearing loss [61]. Another preclinical study evaluated Zucker diabetic fatty rats to determine if the use of losartan could protect against the diabetic damage of the inner ear. This study found that the placebo-treated diabetic rats had worse hearing thresholds and significant changes to the morphology of the intermediate cells of the stria vascularis compared to the losartan-treated diabetic rats [62]. In a study of rats exposed to ototoxic kanamycin, co-administration of the ARB telmisartan resulted in lower hearing threshold shifts and decreased loss of cochlear outer hair cells and spiral ganglion neurons compared with kanamycin exposure alone [63]. As a correlate to the clinical observations in patients with vestibular schwannoma discussed above, researchers employed a mouse schwannoma model to study the impact of losartan treatments, and found significant improvements in blood flow and oxygenation, decreased inflammation, and preserved hearing as compared to the control condition [59]. Taken together, the preponderance of clinical and preclinical data points to modulation of angiotensin signaling as a promising therapy for hearing loss. However, there are significant limitations to current evidence, including a lack of randomized controlled trials, dependence on retrospective analyses subject to biases, heterogeneity in outcome measures across studies, relatively short follow-up periods in most reports, and inconsistency with dosing, timing, and patient selection criteria. These limitations identify the need for well-designed prospective studies before RAS modulation can be recommended as a standard treatment for hearing loss.

## 3. Tinnitus

### 3.1. Epidemiology of Tinnitus

Tinnitus is defined as the perception of noise often described as a “ringing” sound without the presence of an external stimulus. An estimated 50 million Americans reported experiencing any tinnitus, and 16 million reporting experiencing tinnitus at least once daily [64]. Of those that experience tinnitus, 2% report that the symptoms are severe enough to cause significant distress [65]. It is commonly linked with depression, anxiety, and insomnia [66]. Prevalence is higher among older adults, hypertensive patients, smokers, veterans, patients with traumatic brain injury, noise-exposed workers, and musicians [67,68,69,70,71]. Some degree of hearing loss is associated with tinnitus in more than 90% of cases [72]. Other contributing factors include noise exposure, Meniere’s disease (MD), ototoxic medications, middle-ear infections, otosclerosis, vascular malformations, tumors, middle ear spasms, perilymphatic fistula, or head and neck injuries [73,74,75,76,77]. Traumatic brain injury (TBI) affects 1–2 million Americans each year and is a leading war-related injury. In veterans with TBI, there is an increased likelihood of experiencing severe tinnitus compared to those without a history of TBI. Some cases may be complicated by ototoxic medications used for trauma-related infections [76]. Aminoglycosides and chemotherapeutics are known ototoxic medications that can also lead to tinnitus, which can be irreversible [73]. Gentamicin is the most cochleotoxic, followed by tobramycin and amikacin. Cisplatin produces the most severe chemotherapeutic ototoxicity, with fluorouracil and bleomycin also implicated.

Otosclerosis, abnormal bone remodeling of the stapes footplate or otic capsule, can extend into the cochlea and cause tinnitus [75]. Unilateral tinnitus may be a sign of a VS arising from the internal auditory canal or cerebellopontine angle, or a Chiari malformation, where low-lying cerebellar tonsils place tension on the auditory nerve [77].

Somatic tinnitus is associated with temporomandibular joint dysfunction, whiplash, or cervical spine disorders, likely through disinhibition of the dorsal cochlear nucleus [78,79,80].

Usually caused by barotrauma or TBI, perilymphatic fistula involves rupturing of the round or oval window membranes, allowing perilymph to leak into the middle ear. These fistulas cause tinnitus in 61–76% of cases and are often accompanied by hearing loss, vertigo, and aural fullness [76].

### 3.2. Pathophysiology of Tinnitus

Three main theories explain the pathogenesis of tinnitus. First, the loss of cochlear input into the central auditory system causes hyperactivity in low-stimulation neural networks [81,82]. Second, the loss of inhibitory neural connections causes a disruption of auditory feedback loops and loss of normal inhibition—mechanistically similar to the phenomenon observed in phantom limb pain [83]. Lastly, the neurotransmitter abnormalities, particularly involving serotonin and GABA, may explain its frequent link to anxiety and depression [83,84,85].

Noise trauma is also strongly associated with high-pitched tinnitus. Acute exposures above 140 dB can damage cochlear hair cells via vascular, metabolic, and chemical injury, causing cell death and auditory nerve degeneration, sometimes without a detectable threshold shift, giving rise to a condition called hidden hearing loss. When more severe, NIHL often has a steep slope, a risk factor for severe tinnitus, and has been shown to drive neuroplastic changes in central auditory structures [86].

### 3.3. Current Treatments for Tinnitus

Currently, treatment strategies for tinnitus are directed towards managing symptoms, as there are no FDA-approved medications for treating tinnitus [87]. Hearing aids or sound generators can help mask tinnitus, and surgically correcting conductive hearing loss may improve symptoms in select patients [88,89]. Cochlear implantation is effective for many patients with severe SNHL, including those with unilateral deafness, and can significantly reduce tinnitus in a large proportion of recipients [90]. In addition to hearing aids or cochlear implantation, a multidisciplinary approach is often recommended to address the psychological symptoms that often accompany tinnitus. Combining tinnitus retraining therapy (TRT) and cognitive behavioral therapy (CBT) can meaningfully improve quality of life. TRT, which uses counseling and noise generators, has shown substantial benefit in many patients [91]. CBT helps individuals modify their psychological response to tinnitus and strengthen coping strategies, with consistent success across trials [92]. Managing comorbidities such as depression or insomnia can improve quality of life. Nortriptyline increased satisfaction in one trial but did not reduce tinnitus severity, and meta-analyses show no conclusive benefit of antidepressants for tinnitus itself [93].

Procedural interventions such as transcutaneous electrical nerve stimulation or transcranial direct current stimulation, intratympanic dexamethasone, acupuncture and repetitive transcranial magnetic stimulation have been found effective in limited cases with controversial outcomes [2,94,95,96].

### 3.4. RAS-Targeted Therapies for Tinnitus

The effects of RAS modulation have shown promising results related to being protective of hearing loss in the preclinical and retrospective studies discussed above; however, there are no studies currently available that have evaluated RAS modulation on tinnitus. Tinnitus is prevalent in over 90% of cases of hearing loss and the underlying mechanisms of oxidative stress and inflammatory cytokines play a role in both hearing loss and tinnitus [97]. We theorize that RAS modulating therapy would be a promising avenue to pursue in efforts to reduce or prevent tinnitus symptoms because of the overlap in the pathophysiologic mechanisms in SNHL and tinnitus.

## 4. Vertigo

### 4.1. Epidemiology of Vertigo

Vertigo, or the perception of motion in the absence of motion, has a variety of underlying etiologies and can be commonly seen with the previously discussed audiovestibular disorders. Benign paroxysmal positional vertigo (BPPV) is the most common form of peripheral vertigo, or vertigo originating from the vestibular system [98]. BPPV presents with acute episodes of vertigo in the absence of hearing loss. The episodes typically last 30 s to a minute and are provoked by postural changes. BPPV is responsible for over half of cases of peripheral vertigo [98]. Of those that are evaluated by healthcare providers for a complaint of vertigo, over 20% will be diagnosed with BPPV [98]. A subset of patients who suffer from BPPV will go on to develop persistent postural-perceptual dizziness (PPPD). PPPD is the most common chronic vestibular disorder in people 30 to 50 years of age and is four times more common in females [99,100]. PPPD is a chronic functional vestibular disorder resulting in unsteadiness, dizziness, or non-vertiginous dizziness [99]. Although most commonly associated with BPPV, it can also arise in association with vestibular insults such as Meniere’s disease, vestibular neuritis, or vestibular migraine as well as metabolic conditions, allergies or psychological distress [99]. Distinguishing PPPD from other vestibular disorders can be difficult but doing so is essential given the differences in treatment strategies.

Meniere’s disease (MD) is characterized by episodic vertigo, fluctuating low-frequency sensorineural hearing loss, tinnitus, and aural fullness [101]. The prevalence of MD in the United States varies between 3.5 per 100,000 and 513 per 100,000, and more often occurs in older, white, female patients [102].

Acute vertigo seen after a viral illness often falls into one of two categories: labyrinthitis or vestibular neuritis. Labyrinthitis refers to inflammation of the membranous labyrinth within the inner ear, typically resulting from viral or sometimes bacterial infections. There is little epidemiological data, but it is most common in individuals between 40 and 50, and twice as common in women [103]. Vestibular neuritis shares a similar etiology but involves only inflammation of the vestibular nerve and has an estimated annual incidence ranging from 3.5 to 15.5 cases per 100,000 people [104]. Vestibular neuritis is considered the third most common peripheral cause of vertigo, following BPPV and MD [105]. Both conditions can present with acute-onset vertigo, nausea, and vomiting. However, labyrinthitis is distinguished by the presence of auditory symptoms, while vestibular neuritis is limited to vestibular dysfunction alone.

Vestibular migraine is a neurological condition in which vertigo represents the predominant manifestation of a migraine episode [106]. It is the most common cause of both episodic migraines and spontaneous vertigo attacks, with an estimated prevalence of approximately 1% in the general population [107]. There is a 5:1 female-to-male ratio [4]. Patients commonly report acute episodes of vertigo lasting from seconds to several days, often accompanied by dizziness, imbalance, and spatial disorientation [106]. Notably, migraine headache and vestibular symptoms do not consistently occur simultaneously, and there is no predictable temporal relationship between the two [108].

Some less commonly encountered causes of peripheral vertigo include third window syndromes such as superior semicircular canal dehiscence and perilymphatic fistula [109,110,111].

### 4.2. Pathophysiology of Vertigo

The pathophysiology of peripheral vertigo varies based on the subtype. BPPV is thought to arise from calcium-carbonate crystals or otoconia becoming lodged within the fluid-filled semicircular canals of the inner ear [98]. With head movement, the displaced otoconia shifts within the fluid, sending an erroneous stimulus that is perceived as different with respect to the opposite ear leading to the sensation of dizziness [98].

The inner ear contains two distinct fluid compartments—the endolymph and perilymph—with markedly different ionic compositions. The endolymph is characterized by high K^+^ and low Na^+^ concentrations, whereas the perilymph has high Na^+^ and low K^+^ concentrations. The maintenance of these unique ionic environments is critical for inner ear function. Within the cochlea, the stria vascularis secretes K^+^ into the endolymph, while in the vestibular system, analogous vestibular dark cells fulfill a similar role [112]. Ion regulation involves various transporters and channels; basolateral Na^+^/K^+^-ATPases and NKCC1 (Na^+^-K^+^-2Cl^−^ cotransporters) and apical KCNQ1/KCNE1 potassium channels facilitate K^+^ entry into the endolymphatic space [113]. Chloride channels are involved in Cl^−^ recycling to support electrochemical gradients and osmotic balance [114]. Ion transport is also closely linked with water movement, mediated by a range of aquaporin (AQP) channels. AQP2 and AQP3, along with vasopressin-type 2 receptors (V2R), are expressed on the endolymphatic sac and regulate water homeostasis, preventing fluid accumulation [115,116].

Disruption of the inner ear fluid and ionic homeostasis due to genetic mutations illustrates the link between ionic imbalance and vestibular dysfunction and vertigo [117,118,119].

Acquired disturbances, such as trauma or endolymphatic hydrops, can also lead to vertigo through the disruption of ion gradients. MD provides a classic example of this pathophysiologic link. Schuknecht’s theory posits that Meniere’s symptoms arise from a rupture of Reissner’s membrane following endolymphatic hydrops, although in traumatic cases, the rupture may be the initiating event [120]. Mixing of K^+^-rich endolymph with Na^+^-rich perilymph leads to acute depolarization and hyperpolarization of vestibular nerve fibers, triggering vertigo [120]. Also, overexpression of V2R on the luminal epithelium of the endolymphatic sac is seen in MD. This upregulation is hypothesized to lead to subsequent V2R-cAMP-PKA-AQP2 activation and thus endosomal trapping of AQP2, leading to impaired endolymph absorption and hydrops [120,121].

### 4.3. Current Treatments for Vertigo

There are several treatments available for patients with inner ear causes of vertigo, depending on which underlying condition is causing their symptoms. Regarding BPPV, the mainstay treatment is canalith repositioning using maneuvers tailored to the affected semicircular canal, most commonly the Epley maneuver (posterior canal), with alternatives such as the Semont (posterior canal), modified Epley (superior canal), or Barbecue roll (lateral canal) used as indicated [122]. The rationale for these therapies is that using sequential head movements allows for dislodged otoconia to mobilize from the semicircular canal towards the vestibule, which resolves abnormal endolymph flow and stimulation of the cupula, improving vertigo. This is highly effective with a low risk of any serious side effects [123]. Medical therapies have been used for BPPV, but vestibular suppressants are not currently recommended due to a lack of high-quality data supporting their use [122]. If medical therapy is desired, a single dose of antihistamines is preferred over benzodiazepine administration due to their corresponding side effect profiles, including sedation. Medical therapy to reduce subsequent vertigo episodes has been studied, with promising results seen in Vitamin D supplementation being shown to reduce vertiginous spell recurrence [124,125]. Because BPPV is a mechanical disorder caused by displaced otoconia, pharmacologic therapies, including ARBs, have no established role in its treatment.

Treatment of PPPD relies on a multimodal approach designed to alleviate symptoms and improve function. Currently, antidepressants like selective serotonin reuptake inhibitors and serotonin-norepinephrine reuptake inhibitors have been used for relief of dizziness symptoms [126]. Vestibular balance rehabilitation therapy is often utilized to help with balance. CBT has also been shown to improve the efficacy of vestibular physical therapy [99].

MD has a different treatment approach in practice. Clinically, many physicians will use salt restriction or diuretics to treat acute episodes of endolymphatic hydrops, which are a defining component of MD, but not necessarily mutually exclusive to the disease. Recently, there have been multiple systematic reviews highlighting the limited evidence in support of these therapies [127,128]. Other medical therapies have been reported, such as the use of the histamine analog beta-histine. Beta-histine has not been approved for use in the United States but is commonly used in other practices worldwide. However, a multicenter randomized clinical trial did not evidence decreased vertigo spells as compared to placebo [129]. Other adjuvant treatments should also be considered, including treatment of allergies for those who have corresponding symptoms and vestibular rehabilitation, with both being generally well-tolerated [12].

After medical therapies have been evaluated for a patient with MD, procedural options can be considered. Intratympanic corticosteroid injection is commonly used as next-line therapy in practice and is especially effective in patients with comorbid autoimmune disease [130]. Chemical vestibular ablation with intra-tympanic gentamicin may also be used, although it carries a greater risk for hearing loss in the treated ear [12]. If symptoms persist despite these therapies, a decompressive (endolymphatic shunt) or ablative (labyrinthectomy) surgical intervention for the affected ear can be considered to definitively treat persistent, disabling symptoms.

Vestibular neuritis (VN) and labyrinthitis have another treatment paradigm. They are believed to be associated with a viral infection and are often seen after a patient has symptoms of an upper respiratory infection. Methylprednisolone was initially thought to show some benefit in VN, but more recent meta-analysis shows no lasting benefit [131,132]. Corticosteroids may still be considered in labyrinthitis for the treatment of concurrent sudden SNHL if present. For severe acute symptoms, medical management for these conditions is similar to BPPV as discussed above, with a one-time dose of antihistamines being preferred over prescribing benzodiazepines. Vestibular rehabilitation is also strongly recommended and has been shown to have favorable outcomes compared to corticosteroids without the comorbid side effects [133].

### 4.4. RAS Signaling and Inner Ear Ion Homeostasis in Vertigo

Angiotensin II (Ang II) signaling intersects with many of the ion channels and transporters discussed above. AT1R activation is known to stimulate the Na^+^/K^+^/2Cl^−^ cotransporter NKCC1, increasing Cl^−^ uptake and promoting cellular ion retention [134]. Additionally, Ang II is a potent trigger of arginine vasopressin (AVP) release from the brain, and AVP in turn acts on V2R in the endolymphatic sac to regulate aquaporin-2 channels [135]. These synergistic effects with co-activation of AT1 and V2 receptors thus promote increased water retention in endolymph that could be associated with endolymphatic hydrops. Aldosterone, the downstream mineralocorticoid of the RAS, further links Ang II signaling to inner ear ion transport. The epithelium of the endolymphatic sac contains aldosterone-sensitive transport proteins similar to those in the kidney’s distal nephron. These include epithelial sodium channels (ENaC), pendrin (Cl^−^/HCO_3_^−^ exchanger), and Na^+^/K^+^-ATPases, which are highly expressed in both the kidney and inner ear and enhance activity in response to aldosterone, increasing sodium/fluid reabsorption [136,137]. Temporal bone studies have shown that the endolymphatic sac of Ménière’s patients shows histological degenerative changes that reduce expression of mineralocorticoid-regulated Na^+^ transport mechanisms [138]. Patients with MD who are on salt restriction have increased endogenous aldosterone and have been shown to upregulate mRNA for aldosterone-regulated ion transporters in the endolymphatic sac [139].

Cochlear hydrops, characterized by dysregulated endolymphatic fluid homeostasis primarily manifesting as hearing loss, represents another potential target for RAS modulation. Cochlear hydrops commonly presents with fluctuating low-frequency hearing loss, aural fullness, tinnitus, with notably absent vestibular symptoms [140]. Current management strategies, including salt restriction and diuretic therapy, indirectly support a role for vascular and fluid-regulatory pathways in disease pathophysiology. Given evidence that angiotensin signaling influences microvascular tone, inflammation, and tissue remodeling within the stria vascularis, ARB therapy may plausibly affect hydrops-related pathology, though direct clinical evidence is lacking [141].

### 4.5. The RAS and the Blood-Labyrinth Barrier in Vertigo

A lesser-studied contributor of audiovestibular disorders, disruption of the blood-labyrinth barrier (BLB), represents another putative protective target of ARB therapy. Studies show that the breakdown of the BLB has been demonstrated in Meniere’s disease, noise exposure, and age-related hearing loss [142]. Structurally and functionally, the BLB closely resembles the blood–brain barrier (BBB). Although no studies have directly examined RAS modulation on the BLB, there have been several studies evaluating effects of Ang II, AT1R blockade, and AT2R agonism on the BBB. Ang II infusion in mice has been shown to increase BBB permeability and increase leukocyte adhesion of the cerebral microvasculature through oxidative stress [143]. One study evaluated the BBB in rats with spontaneous hypertension, revealing that BBB integrity was maintained in rats treated with the ARB losartan, but not with hydralazine, a direct vasodilator [14]. In a separate study, valsartan was shown to maintain the integrity of the BBB and reduce gap junction proteins diabetic mice as well as suppress expression of the pro-inflammatory cytokines MCP-1 and IL-6 in human brain microvascular cells [144]. Another preclinical study revealed that systemic administration of an AT2R agonist can protect neurons suffering from ischemic—like injury and reduce cortical infarct volumes in rat models [145]. Should AT1R blockade similarly preserve BLB integrity, the effects of promoting maintenance of the inner ear ionic gradient, preserving microvascular blood flow, decreasing immune infiltration and activation, and reducing oxidative stress could provide the foundation for its otoprotective effects. This is especially relevant for MD, where microvascular dysfunction and endolymphatic drops play important roles in disease pathology. The preservation of BLB integrity through ARB therapy could theoretically reduce the inflammatory cascade and fluid dysregulation that trigger vertiginous spells.

## 5. Repurposing RAS-Targeted Therapies for Audiovestibular Disorders

### 5.1. RAS as a Therapeutic Target

The classical RAS is a tightly regulated endocrine cascade that plays a central role in maintaining cardiovascular homeostasis, particularly through the regulation of blood pressure, fluid volume, and electrolyte balance [146]. The classical understanding of the pathway begins in the liver, where hepatocytes synthesize and release angiotensinogen, a large α2-globulin protein that circulates in the plasma. In response to stimuli such as reduced renal perfusion pressure, sympathetic nervous system activation (via β1-adrenergic receptors), or decreased sodium delivery to the distal tubule, the juxtaglomerular cells of the kidney secrete renin. Renin cleaves angiotensinogen to produce angiotensin I (Ang I), a decapeptide with minimal biological activity. Ang I is then converted to the potent octapeptide angiotensin II (Ang II) by angiotensin-converting enzyme (ACE), which is predominantly expressed on the luminal surface of endothelial cells, especially in the pulmonary vasculature [147].

Ang II promotes inflammation when binding to the angiotensin type 1 receptor (AT1R). Once bound to AT1R, there is an upregulation of interleukin-8 (IL-8), select chemokines (MCP-1), cytokines (IL-6, TNF-α, and IL-17A), and adhesion molecules through the NF-κB pathway [148]. Ang II also induces mitochondrial oxidative stress through downregulation of SIRT3 and SIRT6, which leads to hyperacetylation of SOD2 and reactive oxidative species (ROS) production in the mitochondria. Further, Ang II activates NOX2 to produce O_2_^−^, which forms NO_3_^−^ from NO. NO_3_^−^ interrupts the respiratory chain and promotes ROS and O_2_^−^ production in the mitochondria [149].

Ang II is the principal effector molecule of the classical RAS and exerts its physiological effects primarily through binding to the angiotensin II type 1 receptor (AT1R), a G protein-coupled receptor widely expressed in vascular smooth muscle, the adrenal cortex, kidneys, and the central nervous system. Upon AT1R activation, Ang II induces systemic vasoconstriction, stimulates aldosterone secretion from the adrenal zona glomerulosa (promoting sodium and water retention), enhances sympathetic outflow, and increases antidiuretic hormone (ADH) release. These effects collectively raise systemic vascular resistance and blood volume, thereby increasing arterial blood pressure [150]. Additionally, Ang II promotes cellular proliferation, hypertrophy, fibrosis, inflammation, and oxidative stress, which are relevant to its role in pathophysiological conditions such as hypertension, heart failure, and chronic kidney disease [151]. This classical understanding of the RAS has underpinned decades of therapeutic strategies targeting hypertension and cardiovascular disease, primarily through the use of angiotensin converting enzyme inhibitors (ACE-i), angiotensin receptor blockers (ARBs), and direct renin inhibitors [151].

Initially, the RAS was believed to operate solely as a systemic mechanism without tissue specificity. This view shifted when it was discovered that various organs, including the heart, kidney vasculature, pancreas, retina, and brain, can locally synthesize RAS components [152,153,154,155,156,157,158]. This enables the generation of peptides that act in autocrine, paracrine, or even endocrine manners depending on their site of action [159,160].

### 5.2. The Protective Arm of the RAS: AT2R, Mas Receptor, Ang-(1-7), ACE2, and Alamandine

The discovery of additional components of the RAS, including the Mas receptor (MasR), AT2 receptor (AT2R), the angiotensin-(1-7) (Ang-(1-7)) peptide, and the ACE2 enzyme, has expanded the understanding of the RAS beyond its traditional role in vasoconstriction [161]. The ACE2/Ang-(1-7)/Mas receptor axis is widely expressed in reproductive tissues and exerts vasodilatory, anti-inflammatory, antifibrotic, and hormone-regulating effects, supporting processes such as ovulation, implantation, placental function, spermatogenesis, and erectile function [162]. This receptor axis has also been implicated in ovarian pathologies like PCOS, ovarian hyperstimulation syndrome, and ovarian cancer [163].

The discovery of ACE2 as a homolog of ACE marked a pivotal advancement in our understanding of the RAS. Previous studies have demonstrated that ACE2 functions as a carboxypeptidase that cleaves Ang I to generate angiotensin-(1-9), a precursor to the vasodilatory peptide Ang-(1-7) [164,165]. Additionally, ACE2 converts Ang II into Ang-(1-7), with a catalytic efficiency approximately 400-fold greater than it converts Ang I to Ang-(1-9). This highlights ACE2’s strong preference for degrading Ang II to generate the vasodilatory peptide Ang-(1-7) [166].

The AT2R also plays an important role in the protective arm of the RAS. Unlike the AT1R, which mediates vasoconstriction, inflammation, and fibrosis, AT2R activation promotes vasodilation, anti-inflammatory signaling, anti-fibrotic responses, and neuroprotection [167]. AT2R expression is upregulated in response to tissue injury and may enhance its own expression, suggesting a self-amplifying protective role in damaged tissues [168]. Although AT2R stimulation does not significantly lower blood pressure, it has demonstrated therapeutic potential in reducing organ damage and inflammation in models of cardiovascular, renal, and neurological diseases [168]. In a preclinical study using the AT2R receptor agonist Compound 21, diabetic mice were found to have substantially inhibited expression of proteins involved in oxidative stress, inflammation and fibrosis [169].

Ang-(1-7) exerts its vasodilatory, anti-inflammatory, and antifibrotic effects through the MasR, a G protein-coupled receptor expressed in the heart, kidney, brain, vasculature, lung, and testis [170]. The therapeutic potential of the ACE2/Ang-(1-7)/Mas receptor axis is being explored through recombinant ACE2, Mas agonists, and Ang-(1-7) analogs [171]. Alamandine is a newly identified heptapeptide that is structurally and functionally similar to Ang-(1-7) that acts on different G-protein coupled receptor [172]. It can either be generated from angiotensin A via ACE2 or from Ang-(1-7) through decarboxylation. Oral administration of alamandine in hypertensive rats demonstrated sustained blood pressure reduction and cardioprotective effects, highlighting its therapeutic potential as a novel RAS modulator [173].

These protective arms of the RAS, such as ACE/Ang II/AT2R and ACE2/Ang-(1-7)/MasR signaling pathways, have yet to be explored in the context of otoprotection research. Given the substantial mechanistic overlap of the protection afforded by these signaling cascades that is described in other end organ tissues and common audiovestibular pathophysiology, future studies implementing treatments such as AT2R agonists, ACE2 activators, or Ang-(1-7) mimetics hold great promise.

## 6. Conclusions

**Figure 2:**
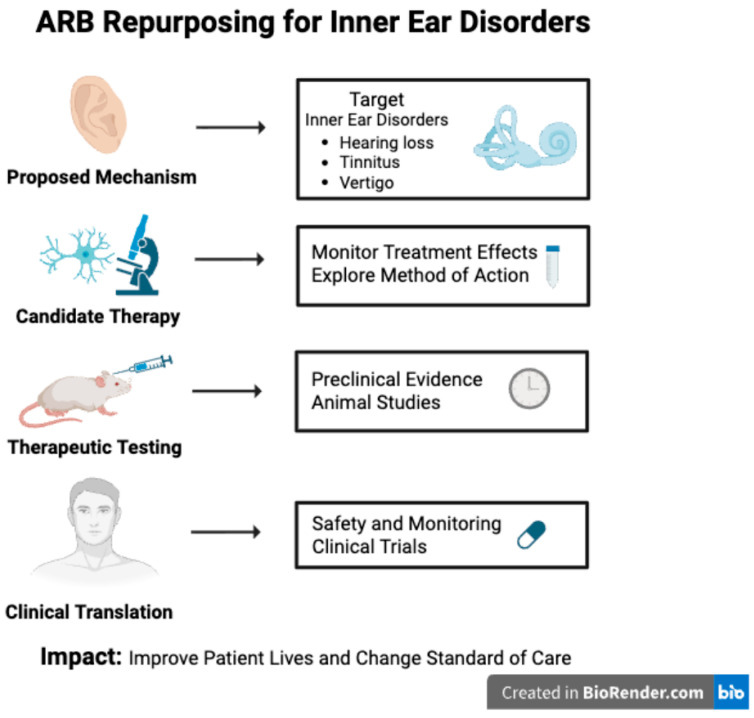
Schematic overview of the repurposing of angiotensin receptor blockers (ARBs) for inner ear disorders. Through enhanced discovery of renin–angiotensin system (RAS) signaling within the auditory system and advancements in preclinical and clinical research, ARBs may be able to provide a new management option for those suffering from inner ear disorders. Created in BioRender. Duffy, C. (2026) https://BioRender.com/xwhxm02.

The protective effects of ARB therapy have been demonstrated in other organ systems through reductions in inflammation and oxidative stress, as well as preservation of microvascular integrity, including at the blood–brain barrier. These effects may similarly extend to the inner ear, which experiences comparable pathogenic mechanisms in audiovestibular disorders. Current evidence supports a protective role for ARBs in preventing hearing loss, likely through their anti-inflammatory and antioxidant properties. Despite these findings, clinical evidence supporting the use of ARB therapy for the treatment of tinnitus or vestibular disorders remains limited. Current associations are largely mechanistic or observational, and no randomized clinical trials have established efficacy for tinnitus, vestibular neuritis, or related vestibular conditions. Further prospective studies are required to define whether modulation of the RAS has a clinically meaningful role beyond hearing preservation. Given their established safety, affordability, and efficacy in diseases such as hypertension and diabetic nephropathy, further well-designed preclinical and clinical trials are needed to optimize ARB selection, dosage, and treatment duration for various audiovestibular disorders (Figure 2). Such studies should also aim to explain the precise molecular mechanisms by which ARBs confer protection within the cochlea, including their effects on oxidative stress, inflammation, and vascular integrity. Establishing standardized treatment parameters and identifying patient populations most likely to benefit will be critical for successful clinical translation.

## Figures and Tables

**Table 1:** Comparison of hypertensive treatment regimens and progression to cochlear implantation among >33,000 patients in the *All of Us* database. ACE-i (angiotensin converting enzyme inhibitor), ARB (angiotensin receptor blocker).

Hypertensive Treatment Regimen	Percentage of Patients Who Underwent Cochlear Implantation (CI)
ACE-i/ARB	6.4 × 10^−4^
Beta blocker/calcium channel blocker	9.7 × 10^−4^
Chi-square	0.961
*p*-value	0.16
Relative Risk Reduction	34.42%

## Data Availability

This study used data from the *All of Us* Research Program’s Registered Tier Dataset [version 7], available to authorized users on the Researcher Workbench.

## Abbreviations

The following abbreviations are used in this manuscript:

RAS	Renin–angiotensin system
ARBs	Angiotensin II type 1 receptor blockers
SNHL	Sensorineural hearing loss
PPPD	Persistent postural-perceptual dizziness
VS	Vestibular schwannoma
NIHL	Noise-induced hearing loss
SSNHL	Sudden sensorineural hearing loss
AIED	Autoimmune inner ear disease
ROS	Reactive oxidative species
RNS	Reactive nitrogen species
NF-κB	Nuclear factor kappa B
PPARγ	Peroxisome proliferator-activated receptor γ
HPD	Hearing protection devices
CROS	Contralateral routing of sound
MD	Meniere’s disease
TBI	Traumatic brain injury
TRT	Tinnitus retraining therapy
CBT	Cognitive behavioral therapy
BPPV	Benign paroxysmal peripheral vertigo
AQP	Aquaporin
V2R	Vasopressin-type 2 receptors
VN	Vestibular neuritis
Ang II	Angiotensin II
AVP	Arginine vasopressin
ENaC	Epithelial sodium channels
Ang I	Angiotensin I
ACE	Angiotensin-converting enzyme
AT1R	Angiotensin II type 1 receptor
ADH	Antidiuretic hormone
ACE-i	Angiotensin-converting enzyme inhibitor
MasR	Mas receptor
AT2R	Angiotensin II type 2 receptor
Ang-(1-7)	Angiotensin-(1-7)
Ang-(1-9)	Angiotensin-(1-9)
ACE2	Angiotensin-converting enzyme type 2
BLB	Blood–labyrinth barrier
BBB	Blood–brain barrier
